# Supplementary material for: Association between oral microbiome and seven types of cancers in East Asian population: a two-sample Mendelian randomization analysis
Source: Front Mol Biosci. 2023 Nov 21;10:1327893. doi: 10.3389/fmolb.2023.1327893 (PMC10702768; doi:10.3389/fmolb.2023.1327893)
Supplement: Supplementary file 1 [file DataSheet1.ZIP › Supplementary Materials/MR plots of saliva/saliva═╝/Lung Cancer/pheno.662_to_lung cancer_leave_one_out.pdf]

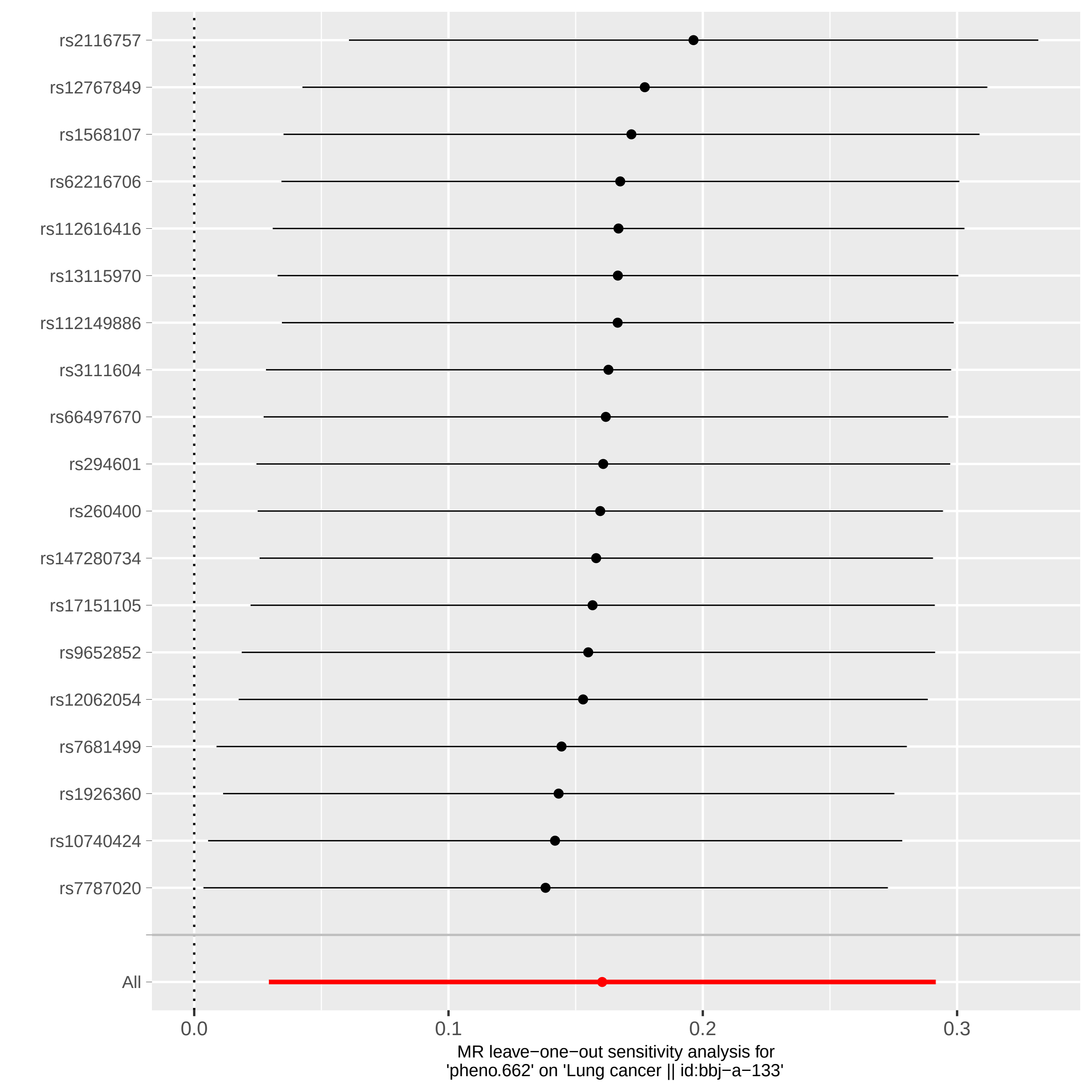
rs2116757
rs12767849
rs1568107
rs62216706
rs112616416
rs13115970
rs112149886
rs3111604
rs66497670
rs294601
rs260400
rs147280734
rs17151105
rs9652852
rs12062054
rs7681499
rs1926360
rs10740424
rs7787020
All
0.0
0.1
0.2
0.3
MR leave−one−out sensitivity analysis for
'pheno.662' on 'Lung cancer || id:bbj−a−133'
